# A Finite Element Model for Simulating Stress Responses of Permeable Road Pavement

**DOI:** 10.3390/ma17123012

**Published:** 2024-06-19

**Authors:** Jhu-Han Siao, Tung-Chiung Chang, Yu-Min Wang

**Affiliations:** 1 Department of Civil Engineering, National Pingtung University of Science and Technology, Pingtung 91201, Taiwan; ggg49867531@gmail.com; 2 Department of Civil Engineering and Geomatics, Cheng Shiu University, Kaohsiung 83347, Taiwan; 4946@gcloud.csu.edu.tw; 3 General Research Service Center, National Pingtung University of Science and Technology, Pingtung 91201, Taiwan

**Keywords:** permeable road pavement, dynamic load experiment, finite element model, stress responses, climate change

## Abstract

Permeable road pavements, due to their open-graded design, suffer from low structural strength, restricting their use in areas with light traffic volume and low bearing capacity. To expand application of permeable road pavements, accurate simulation of stress parameters used in pavement design is essential. A 3D finite element (3D FE) model was developed using ABAQUS/CAE 2021 to simulate pavement stress responses. Utilizing a 53 cm thick permeable road pavement and a 315/80 R22.5 wheel as prototypes, the model was calibrated and validated, with its accuracy confirmed through *t*-test statistical analysis. Simulations of wheel speeds at 11, 15, and 22 m/s revealed significant impact on pavement depths of 3 cm and 8 cm, while minimal effects were observed at depths of 13 cm and 33 cm. Notably, stress values at a depth of 3 cm with 15 m/s speed in the open-graded asphalt concrete (OGFC) surface layer exceeded those at the speed of 11 m/s, while at a depth of 8 cm in the porous asphalt concrete (PAC) base layer, an opposite performance was observed. This may be attributed to the higher elastic modulus of the OGFC surface layer, which results in different response trends to velocity changes. Overall, lower speeds increase stress responses and prolong action times for both layers, negatively affecting pavement performance. Increasing the moduli of layers is recommended for new permeable road pavements for low-speed traffic. Furthermore, considering the effects of heavy loads and changes in wheel speed, the recommended design depth for permeable road pavement is 30 cm. These conclusions provide a reference for the design of permeable road pavements to address climate change and improve performance.

## 1. Introduction

Many researchers have demonstrated that permeable road pavements with high porosity offer numerous advantages. These include reducing flood runoff and improving hydrology [1,2], reducing noise [3,4,5], aiding the growth of ecological environment plants [6], and lowering surface temperatures and the urban heat island effect [7]. However, their open-graded design makes them susceptible to low structural strength and clogging issues [8,9,10], restricting their application to areas with light traffic volume and low bearing capacity [8,11,12]. Thus, for the effective implementation of permeable road pavements, the accuracy of mechanical parameters is crucial in designing them using a mechanistic procedure. Accurately predicting these parameters based on traffic and environmental data is essential for designing more durable permeable road pavement structures in the future [13].

Various methods are employed to study the mechanical responses of pavements, include field testing, theoretical analysis, and FE simulation. Field testing provides real-time insights into pavement behavior [14,15,16] but demands significant time and financial resources [17,18]. Multi-layer elastic analysis, while cost-effective and straightforward, may be limited in analyzing complex environments and load conditions due to its inherent assumptions [19,20]. To overcome this limitation, the 3D FE method is utilized. The 3D FE model can easily handle complex scenarios, considering static and dynamic loads, material elasticity, viscoelastic behavior, and bonding between pavement layers. It also enables simulation of the distribution of internal mechanical responses of pavements, making it an effective tool for studying pavement behavior [18,19].

Over the past 20 years, numerous researchers have utilized ABAQUS software to develop 3D FE models for simulating the internal mechanical responses of pavements [15,17,18,20]. These models have been validated for their effectiveness and reliability based on experimental data. Dynamic analysis using 3D FE models has been found to provide temporally correlated histories of pavement response, making them more comparable to field measurements [18].

Through a comprehensive review of past research, it is evident that factors such as pavement thickness, vehicle speeds, and loadings significantly influence the internal mechanical response of pavements. Selsal et al. [17] investigated changes in the mechanical performance of asphalt pavements through numerical, experimental, and parametric processes to assess the influence of pavement thickness and modified asphalt mixtures. They found that increasing asphalt thickness led to a decrease in maximum strain, nearly halving as pavement thickness doubled. Assogba et al. [13] utilized 3D FE modeling and implicit dynamic analysis to study the effects of moving axle loads on semi-rigid pavements. Their results showed that lower traffic speeds and additional weight extensions on truck axles had adverse effects on mechanical parameters. Wang and Li [21] developed a 3D FE model for flexible pavements and investigated moving vehicle load conditions. They found that vehicle speeds and pavement depths significantly influenced the duration of pressure pulses in the asphalt layer. Al-Qadi et al. [22] established transient dynamic load models using 3D FE models, finding that the rate of rutting damage in the pavement structure depends on vehicle speeds and pavement temperatures.

A review of the literature reveals that while most studies are dedicated to simulating the mechanical behavior of impervious pavements, there is a relatively limited number of studies focused on permeable road pavements. Thus, it is necessary to conduct in-depth research to simulate the mechanical responses of permeable road pavements. Additionally, understanding the effect of moving loads from heavy traffic and varying speeds on mechanical responses of permeable road pavements with different thicknesses is a critical step toward expanding the use of permeable road pavements.

## 2. Study Objective

This study aims to evaluate the effect of a moving heavy load and varying traffic speeds on the mechanical responses at different depths of permeable road pavement. The study combines 3D FE simulations with a dynamic load experiment to analyze the internal dynamic responses of the pavement. ABAQUS software was utilized to establish a 3D FE model of the permeable road pavement. The model prototypes were based on a permeable road pavement from Ke-Da Road in Pingtung, Taiwan, and a single wheel of a 35-ton truck used in the dynamic load experiment. The stress responses of the 3D FE model were calibrated using the stress responses from the dynamic load experiment to ensure model feasibility. Subsequently, the validated 3D FE model was employed to simulate the stress responses of the permeable road pavement for different wheel traveling speeds. 

## 3. Materials and Methods

### 3.1. Permeable Road Pavement

**Figure 1:**
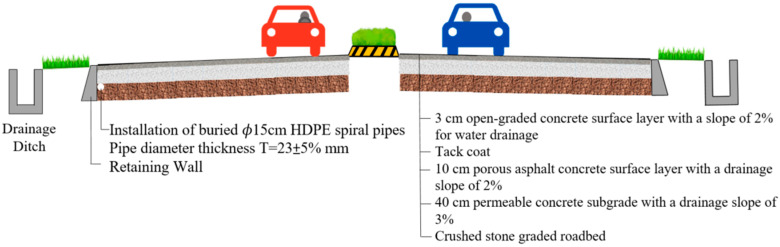
Road standard cross-section diagram (S = 1/100).

The study area for the permeable road pavement is located in Section 3 of Ke-Da Road, spanning from km 15k + 125 to km 15k + 225, along Line 187 in Pingtung Country, Taiwan. This section of the road is a dual carriageway with a 3 m lane for rapid vehicles and a 5.5 m lane for mixed vehicles, running in both north and south directions. As illustrated in Figure 1, the pavement structure comprises a 3 cm OGFC surface layer, a 10 cm PAC surface layer, and a 40 cm permeable concrete (PC) base layer. Both the surface and base layers have a drainage slope of 2%, while the subgrade has a drainage slope of 3%.

In this study, the study area corresponds to that used by Hsing et al. [14]. The maximum nominal aggregate size of the OGFC layer in the permeable road pavement is 9.5 mm, with a porosity of 16.4%. The OGFC layer contains a significant amount of single-size aggregates to create higher porosity, allowing water to flow freely between continuous voids and drain rainwater quickly to the outer edge of the road. The larger inter-particle voids in the permeable road pavement increase surface roughness, providing better frictional resistance to enhance driving safety [23,24]. Similarly, PAC also consists of material with high porosity and a high proportion of coarse aggregates. PAC has a maximum nominal aggregate size of 19 mm, with a porosity is 17.1%. PAC allows water to quickly infiltrate and drain through a large number of voids in the pavement. 

PC is composed of cement, coarse aggregates, and water for mixing. The United States Environmental Protection Agency [25] considers PC as the optimal material for controlling initial stormwater pollution and management. PC can reduce the runoff volume in paved areas, decrease the need for stormwater detention ponds, and lower the required capacity of rainwater drainage pipes. Additionally, it provides filtration capabilities, resulting in a reduction in pollutants entering rivers and reservoirs [26]. Therefore, rainwater can be effectively managed using PC, which improves groundwater quality through the process of water infiltration. The material properties of the surface and base layers of the permeable road pavement are presented in Table 1.

### 3.2. Pavement Instrumentation

**Figure 2:**
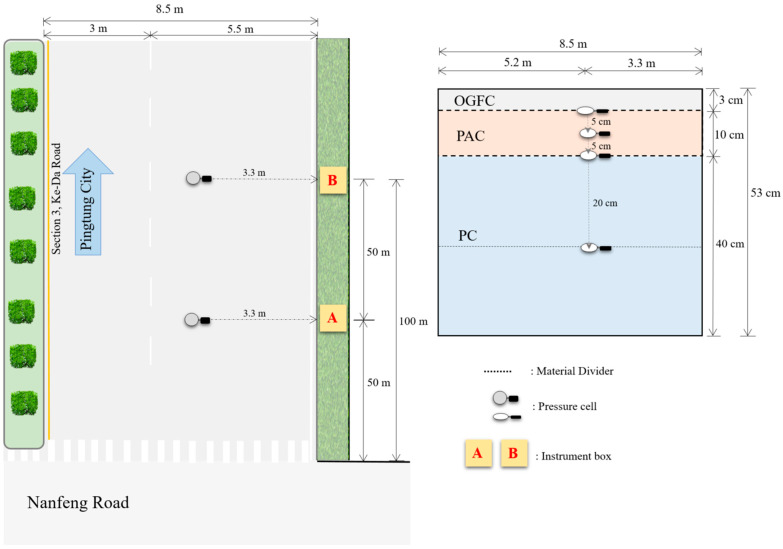
Pressure cell locations in permeable road pavement (not in scale).

**Figure 3:**
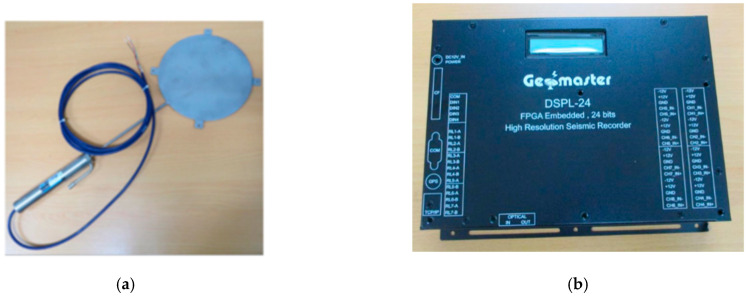
The instrument and equipment used in the test. (**a**) Pressure cell and (**b**) high-resolution seismic recorder DSPL-24.

Location A and Location B were selected within the experimental area during the road construction period in 2018, as illustrated in Figure 2. To monitor stress responses of the permeable road pavement, Model 3500 pressure cells (Figure 3a) from Geokon, Inc. (Lebanon, NH, USA) were embedded at depths of 3 cm, 8 cm, 13 cm, 33 cm, and 53 cm. All pressure cells were embedded approximately 3.3 m from the edge white line in the mixed lane. During the dynamic load experiment, stress data from the pressure cells embedded in the permeable road pavement were collected using the high-resolution seismic recorder DSPL-24 (Figure 3b). The DSPL-24 recorded data at a rate of 50 samples per second.

Pressure cells, sometimes called total pressure cells or total stress cells, are designed to measure total stresses in earth or rock fills and embankments. They respond not only to soil pressures but also to groundwater pressures or pore water pressure. Typical applications of pressures cells include measuring traffic-induced stresses on roadway subgrades, airport roadways, or under railroad tracks. In this study, pressure cells were installed with the flat surfaces horizontal to measure stresses of the permeable road pavement. Formula (1), as follows, was used for converting readout voltages to pressure:

(1) $$P=(R_{1}-R_{0})G\times 10/V_{1},$$

where

P is the applied pressure, kPa;
$R_{1}$ and $R_{0}$ are the current and initial output readings in millivolts, volts, or milliamps;
G is the gage factor from an instruction manual of Geokon, Inc. [27];
$V_{1}$ is input voltage, 10 VDC.

### 3.3. Dynamic Load Experiment

**Figure 4:**
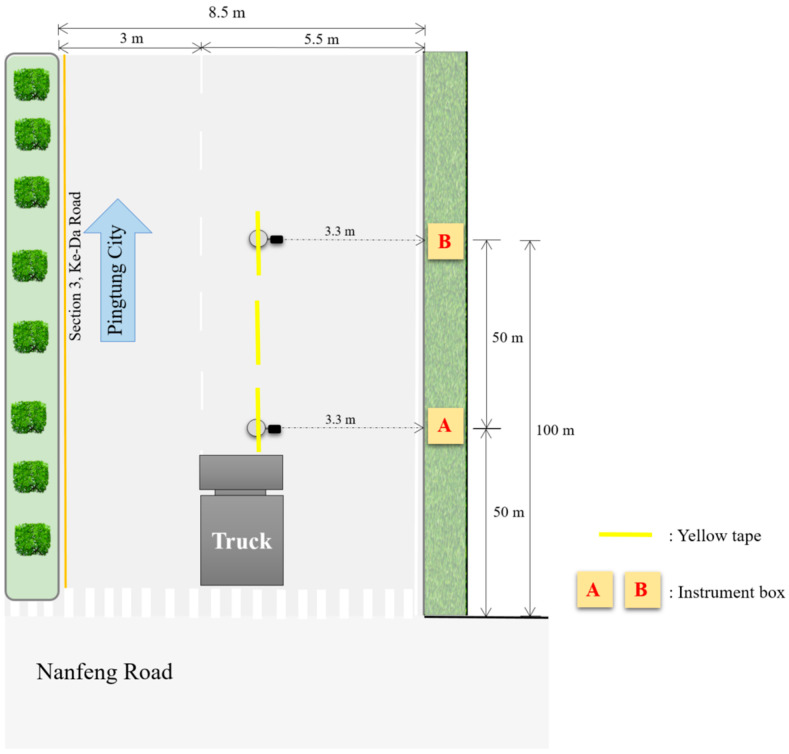
Top-view diagram of the dynamic load experiment.

In this study, a dynamic loading experiment was conducted on 25 October 2019, during a sunny day. A 35-ton semitrailer truck with a four-axle configuration from Seven Seas Transportation Company, Pingtung, Taiwan, was used as the experimental vehicle to investigate the stress behavior of the permeable road pavement under heavy vehicle loading, as shown in Figure 4.

Before the experiment, the instrument locations were marked with yellow tape, and trajectory lines were set at a distance of 3.3 m from the edge of the road to guide the path of the truck during the experiment. During the experiment, the truck was driven at speeds of 40 km/h, 60 km/h, and 80 km/h within the experimental area and repeated at least three times to ensure data integrity. However, it is easier to accurately apply pressure to the instrument during low-speed truck operation. The stress results obtained at a speed of 40 km/h from dynamic load experiment are used to verify the stress results of the 3D FE model.

To ensure the accuracy of the stress results, the data obtained at a speed of 40 km/h from the dynamic load experiment were further processed. By referring to the data processing method in the previous study by Hsing et al. [14], the acceptable data were extracted. The data processing steps are as follows:

1. Four peaks in the stress of the permeable road pavement must be detected by the pressure cells. The four peaks indicate that four wheels pressed against the instrument;
2. The detected values must show positive pressures;
3. The stress response of the surface layer must be larger than those of the base layer;
4. The time difference between the two axles of the truck should be calculated to determine if the trial is acceptable considering the speed.

### 3.4. Finite Element Models

#### 3.4.1. Geometric Model and Material Parameters

**Figure 5:**
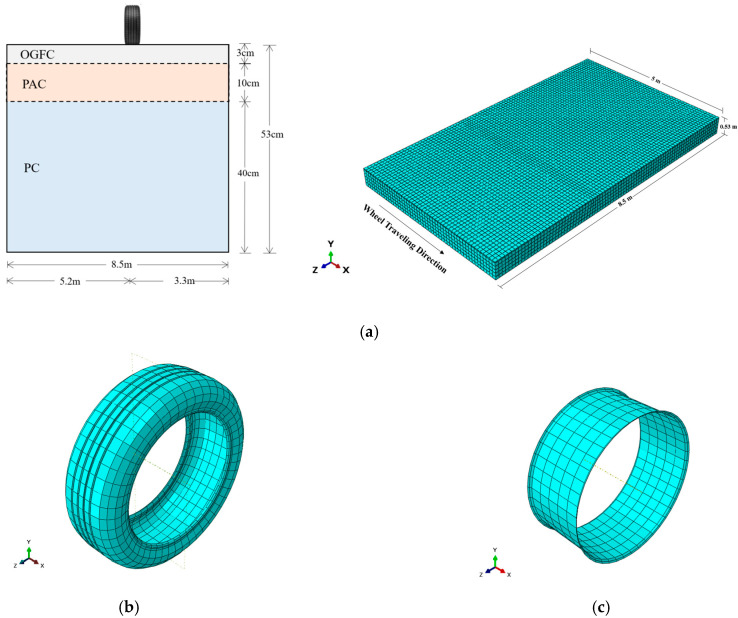
(**a**) Permeable road pavement model; (**b**) the tread and carcass of wheel model; (**c**) rim of wheel model.

This study utilized ABAQUS/CAE 2021 to develop the interaction model. Considering numerical stability and convergence, non-linear dynamic analysis (Abaqus/implicit) was used to simulate the stress responses of the model. The implicit method has better numerical convergence than the explicit method [13]. The interaction model consists of a pavement model and a wheel model. The permeable road pavement on Ke-Da road serves as the prototype for the pavement model, as shown in Figure 5a. The geometric dimensions of the pavement model are 5 m (*x*-axis) × 0.53 m (*y*-axis) × 8.5 m (*z*-axis), where the *x*-axis represents the direction of wheel travel, the *y*-axis represents the depth, and the *z*-axis represents the transverse direction. 

The pavement model is divided into three layers: the OGFC surface layer, the PAC base layer, and the PC base layer. The interfaces between the layers were assumed to be fully bonded. The material parameters set for each layer are shown in Table 2. The materials employed in the 3D FE model were characterized as homogeneous. The elastic moduli and Poisson ratios of the OGFC, PAC, and PC layers were determined using the calibration method described by Ge et al. [28], in order to better match the properties of the actual on-site pavement materials. Detailed instructions are provided in Section 3.4.3. The temperature effect was disregarded, and it was assumed that all layers exhibited linear elastic isotropic behavior. The friction coefficient of the surface layer, obtained from the field skid resistance test, is 7.02. This value was used in the interaction settings in Abaqus.

The wheel of the 35-ton truck serves as the prototype wheel model. The wheel model (see Figure 5b) is based on the standard dimensional data of the 315/80 R22.5 wheel provided by the Michelin Wheel Company (Greenville, SC, USA) [29]. Since this study focuses on the stress response of the pavement, the tread and carcass portions of the wheel were considered to be elastic, while the belt and cord were neglected. The rim is shown in Figure 5c, which was treated as a rigid body. The material dimensions for the wheel model are specified in Table 3. Due to geometric parameters of the wheel being commercial secrets, the geometric parameters of the wheel model in this study were referenced from the research by Lu et al. [19], as listed in Table 4.

#### 3.4.2. Meshing and Boundary Conditions

**Figure 6:**
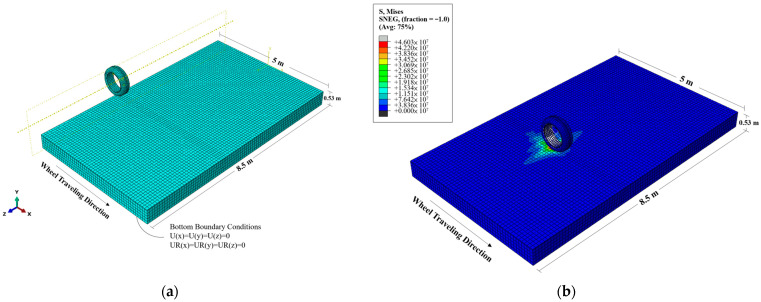
(**a**) Boundary conditions and mesh generation of the wheel–pavement interaction model. (**b**) Stress responses in the model.

The selected element type for meshing in this study is the Continuum 3D 8-node linear brick elements with reduced integration (C3D8R), chosen to enhance the convergence rate and computational efficiency, as shown in Figure 6a. The boundary conditions of the model are as follows: the upper surface and the front and rear surfaces perpendicular to the travel direction are fully free, while the bottom is constrained to zero displacement in the x, y, and z directions, ensuring no vertical or horizontal movement at the base of the pavement model. The hard contact friction model available in ABAQUS was used to simulate the contact behavior between the wheel and the road surface. 

In order to simulate the stress responses of permeable road pavement, the static load and dynamic pressure of the wheel model were set in Abaqus. The single wheel of the first axle of the experimental truck, with a load of 3.5 tons, was used in the modeling. To simulate different scenarios, wheel speeds of 11, 15, and 22 m/s along the traveling track were set. The track width is consistent with the wheel width, which is 0.315 m. In this paper, the stress data of the elements at the middle of surface and base layers were selected for analysis (Figure 6b).

#### 3.4.3. Calibration and Validation Procedure

Since the material parameters of the 3D FE model are obtained from laboratory tests, they may not completely match the material characteristics of the field pavement under traffic loading conditions. Therefore, calibration is necessary after establishing the 3D FE model of the permeable road pavement. Calibration ensures that the numerical values obtained from the model match the measurements obtained from the dynamic load experiment. 

Following the approach proposed by Ge et al. [28], the elastic modulus and Poisson’s ratio of each pavement layer were adjusted in the calibration procedure to better match the elastic properties of the actual field pavement. To accurately determine the elastic properties of each layer, this study utilizes stress data from pressure cells and the 3D FE model at specific pavement depths: 3 cm (bottom of OGFC), 8 cm (middle of PAC), and 33 cm (middle of PC), characterizing OGFC, PAC, and PC, respectively. However, the surface layer, which directly contacts the moving wheel, is prone to damage and exhibits significant variations in its elastic properties. Thus, it is necessary to determine the elastic properties of the subgrade first, followed by the base layer, and finally the surface layer. By varying the moduli and Poisson ratios of the subgrade, base layer, and surface layer in elastic properties of the 3D FE model, the stress curves obtained from the measurement and the simulation are fitted to each other as closely as possible, with a focus on matching the peak values and shapes. Following a series of calibration and validation processes, the elastic modulus and Poisson’s ratio of each layer were determined, as listed in Table 2.

#### 3.4.4. Statistical Analysis and Prediction Error

To compare simulated and measured stresses, *t*-tests were conducted. The null hypothesis (H_0_) assumes no difference between the simulated and measured stress values, while the alternative hypothesis (H_1_) suggests a difference between them. A significance level of α = 0.05 was set for the analysis. If the *p*-value exceeds 0.05, the difference is considered statistically nonsignificant. 

Moreover, referring to Assogba et al.’s study [13], the prediction error ($e_{r}$) for simulating the stress response of the permeable road pavement was calculated to evaluate the effectiveness of the established 3D FE model, using Formula (2):

(2) $$e_{r}=(\frac{S_{s}-S_{m}}{S_{m}})\times 100,$$

where

$S_{s}$ is the simulating stress, MPa;
$S_{m}$ is the measured stress, MPa.

A negative $e_{r}$ value signifies an underestimated stress from 3D FE simulations, whereas a positive $e_{r}$ value indicates an overestimated stress.

## 4. Results and Discussion 

### 4.1. Stress Distribution in the Permeable Road Pavement from Dynamic Load Experiment

**Figure 7:**
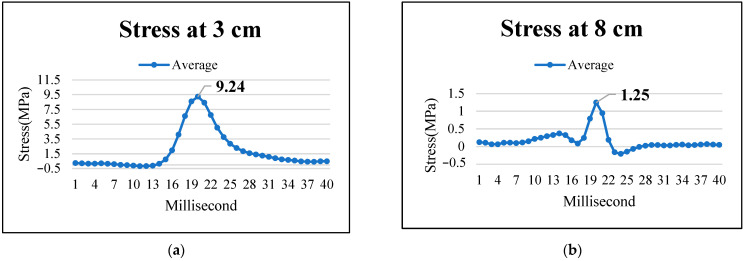

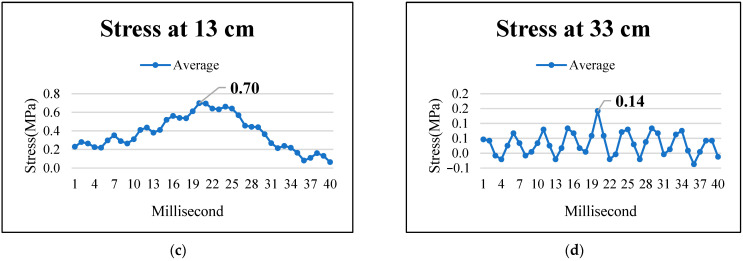
Stress–time curves at the following depths of the permeable road pavement: (**a**) 3 cm, (**b**) 8 cm, (**c**) 13 cm, and (**d**) 33 cm.

In this section, the stress distributions at various depths of the permeable road pavement during the dynamic load experiment at a truck speed of 40 km/h were investigated. As illustrated in Figure 7a,b, there is a progressive rise in stress values as the wheel approaches the pressure cell. Notably, the stress peaks when the wheel is directly aligned with the pressure cell and gradually diminishes as the wheel moves away from the instrument, eventually returning to zero over time. Furthermore, the stress–time curve exhibits an asymmetric shape, consistent with the findings of Imjai et al. [30] and Sun et al. [31].

The maximum stresses at pavement depths of 3 cm and 8 cm were approximately 9.2 MPa and 1.2 MPa, respectively. However, stresses gradually decreased as the wheel load moved downwards. The stress at depths of 13 cm and 33 cm was approximately 0 MPa, as depicted in Figure 7c,d.

Stresses are highest near the loading area and decrease rapidly with increasing distance from it, consistent with the findings of Liu et al. [15]. The stress in the OGFC surface layer remains significant compared to the PAC base layer, while the stress in the PC base layer is smaller. This is because the modulus of the OGFC is larger than that of the PAC base layer. The OGFC and PAC layers attenuate the wheel load, significantly reducing its impact on the PC layer. Therefore, due to the large deformation occurring in the loading area, it is crucial to investigate the mechanical response of the OGFC and PAC layers.

### 4.2. Comparison of Measured and Simulated Stresses

**Figure 8:**
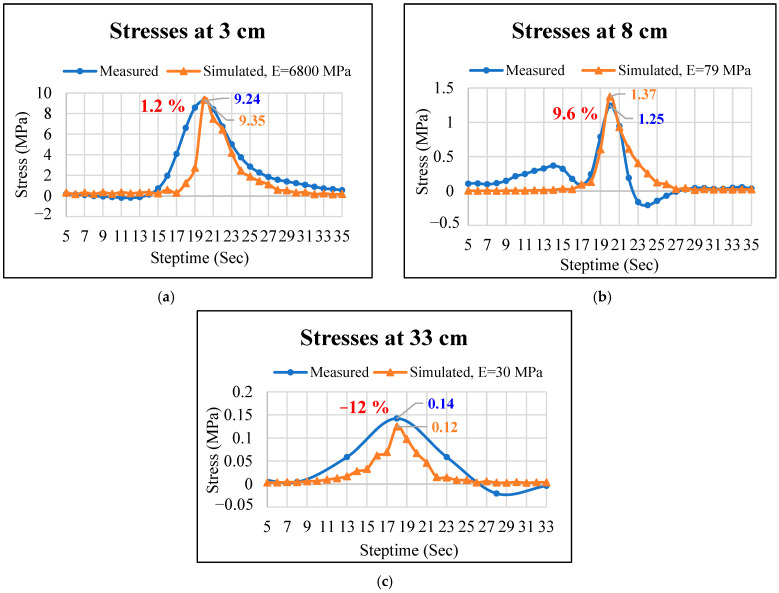
Comparisons between measured and simulated stresses at different depths of permeable road pavement: (**a**) 3 cm, (**b**) 8 cm, and (**c**) 33 cm.

Simulated stresses at different pavement depths at a speed of 11 m/s were used to compare the measured stresses at different pavement depths at a speed of 40 km/h. The comparison results of measured stresses from the dynamic load experiment and simulated stresses from the 3D FE model were obtained through calibration and validation procedures, as shown in Figure 8. From Figure 8a, it can be observed that both the simulated and measured stress–time curves at the depth of 3 cm closely resemble each other, with stress peak values (approximately 9 MPa) being highly comparable. 

From Figure 8b, there is a slight discrepancy in the shape and the peak value between the simulated stress curve and the measured stress curve at the depth of 8 cm. This discrepancy may have arisen because the measured stress is generated by the load applied by the truck wheel on the pavement instrument, while the simulated values are obtained from the load exerted on a single mesh element of the 3D FE model. The result also indicates that the actual test application time is longer [32]. By calculating the prediction error, it is also found that the established 3D FE model predicted the stress peaks observed at depths of 3 cm and 8 cm of the permeable road pavement with a prediction error of less than 10%.

Both the measured and simulated stress peak values at the depth of 33 cm are quite close, approximately 0.1 MPa (see Figure 8c). In the statistical analysis procedure, *t*-tests were used to compare the difference between measured and simulated stresses, the results of which are shown in Table 5. The stress analysis results were *t*(74) = 1.24 (*p* > 0.05), *t*(78) = 0.47 (*p* > 0.05), and *t*(72) = 1.93 (*p* > 0.05) at the depths of 3 cm, 8 cm, and 33 cm, respectively. Therefore, the differences in stresses of layers between measured and simulated are nonsignificant under a 95% confidence interval. In summary, the 3D FE model was validated and considered dependable for subsequent analysis.

### 4.3. Stresses Simulated at Different Speeds

**Figure 9:**
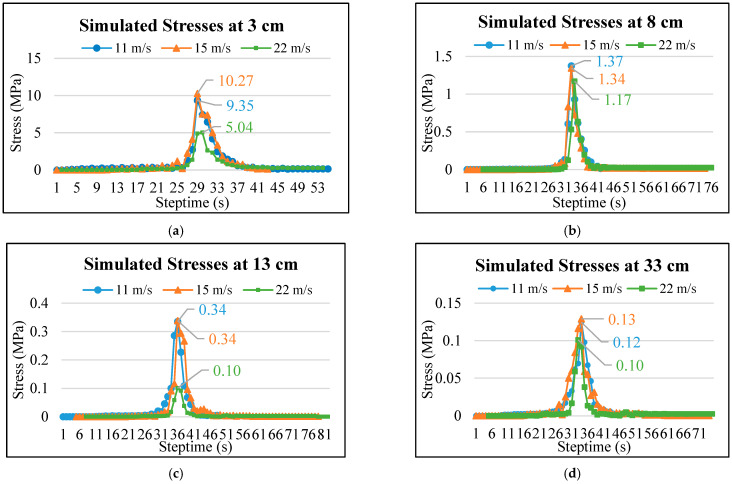
Simulated stress responses at various depths: (**a**) 3 cm, (**b**) 8 cm, (**c**) 13 cm, and (**d**) 33 cm of the permeable road pavement under speeds of 40 km/h, 60 km/h, and 80 km/h.

To simulate stresses at different truck speeds, the wheel model was assumed to move at three constant speeds of 11 m/s, 15 m/s, and 22 m/s on the permeable road pavement model. Figure 9 illustrates the simulated stress results at different depths and wheel speeds from the 3D FE model. Figure 9a shows that the maximum stress at the depth of 3 cm occurs at 15 m/s (10.27 MPa), while the minimum stress occurs at 22 m/s (5.04 MPa). These results reveal that the stress response at the depth of 3 cm in the OGFC layer at the wheel speed of 15 m/s is slightly higher than that at the wheel speed of 11 m/s. There is a marginal increase with the increase in speed, similar to the findings in the research of Al-Qadi et al. [33]. 

Figure 9b shows that the simulated stresses at the depth of 8 cm are 1.37, 1.34, and 1.17 MPa at speeds of 11, 15, and 22 m/s, respectively. The stresses at this depth are slightly higher at the wheel speed of 11 m/s compared to those at 15 m/s. Additionally, the results indicate that the minimum stress occurs at a wheel speed of 22 m/s. Figure 9c presents the maximum simulated stresses at the depth of 13 cm at speeds of 11 and 15 m/s are nearly identical, approximately 0.34 MPa, whereas the simulated stress at this depth is almost 0 MPa at a speed of 22 m/s. However, the effect of wheel speed at a depth of 33 cm has almost no effect, as shown in Figure 9d. 

Overall, the simulated stresses decrease significantly with increasing depths at each speed, particularly when the wheel load is transferred from 3 cm to 8 cm. However, the wheel speeds have almost no effect at the depth of 33 cm in the pavement model. Similar observations were also found in the previous study [13]. It is worth noting that, compared with the underlying PAC and PC base layers, the OGFC surface layer has a higher elastic modulus, resulting in different trends in response to speed variations. According to the FE simulation results of pavement stress by Vijapure et al. [34] and Ahmed et al. [35], the elastic modulus of the material affects the stress responses in the pavement.

**Figure 10:**
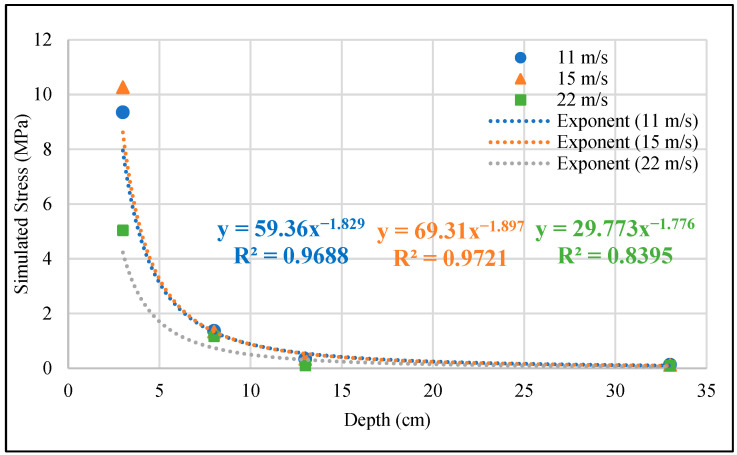
Simulated stress responses at various depths in the permeable road pavement under different wheel speeds.

Figure 10 presents the simulated results at various depths in the permeable road pavement under three wheel speeds. The stress responses decrease with increasing pavement depths at each speed, indicating a negative exponential growth. The stress response at the depth of 3 cm in the permeable road pavement is significantly affected by the wheel load moving at 15 m/s. Special attention is required for the deformation of the OGFC surface layer when the speed is 15 m/s. Lower loading speeds result in increased stress responses and prolonged action times for both the OGFC surface layer and the PAC base layer in the permeable road pavement, negatively impacting pavement performance. Therefore, it is recommended to increase the moduli of the surface and base layers to reduce stress responses in the design and construction of new permeable road pavements intended for low-speed traffic. In addition, it was found that changes in wheel speed have almost no effect at a depth of 30 cm in the pavement. Therefore, it is recommended that the design depth of permeable pavement be 30 cm, which is the same as the conclusions of Yang et al. [7] and Hsing et al. [14].

## 5. Conclusions

The 3D FE model developed in this research for permeable road pavement stress response calculations is more representative, as it can handle dynamic traffic loads and varying speeds, and readily adjust pavement parameters including the elastic properties to better fit the actual conditions. This study presents the stress responses of the permeable road pavement from the dynamic load experiment, which were used to calibrate and validate the accuracy of the established 3D FE model for simulating pavement stress responses under dynamic wheel loads and different speeds. The main conclusions obtained are summarized as follows:

- The stress in the OGFC surface layer is higher than in the PAC and PC base layers due to the OGFC’s larger modulus. The OGFC and PAC layers attenuate the wheel load, reducing its impact on the PC layer. Therefore, it is crucial to investigate the mechanical response of the OGFC and PAC layers due to the large deformation in the loading area.
- The statistical results from *t*-tests indicate that there are nonsignificant differences in the layer stresses between the measured and simulated stress values within a 95% confidence interval. For depths of 3 cm and 8 cm in the permeable road pavement, which are easily affected by loads, it was found that the simulated stresses at these depths were predicted with an error of less than 10% compared to the field-measured stresses. The 3D FE model was successfully validated and considered dependable for subsequent analysis.
- From the simulation results, it was observed that at a depth of 3 cm, the simulated stress values at wheel speeds of 11 m/s and 15 m/s were 9.35 MPa and 10.27 MPa, respectively, indicating an increasing trend in stress simulation. However, at a pavement depth of 8 cm, the simulated stress values at wheel speeds of 11 m/s and 15 m/s were 1.37 MPa and 1.34 MPa, respectively, showing a decreasing trend in the stress simulation. This may be attributed to the higher elastic modulus of the OGFC surface layer, which results in different response trends to velocity changes.
- Lower loading speeds increase stress responses and prolong action times at the OGFC surface layer and PAC base layer, negatively impacting pavement performance. Therefore, it is recommended to increase the moduli of these layers in designing and constructing new permeable road pavements for low-speed traffic. Given that the increase in wheel speed had almost no impact on the simulated stress response at the pavement depth of 33 cm, the recommended design depth of permeable road pavement is 30 cm, considering heavy load and wheel speed changes.

While this study offers noteworthy observations into stress responses of permeable road pavements under dynamic loads, several areas require further investigation. Future research should focus on the decay of mechanical responses under long-term loading, including durability and fatigue. Additionally, the effects of environmental factors such as temperature fluctuations and moisture levels on stress responses and pavement performance need to be explored. Moreover, the impact of varying dynamic load patterns, including traffic densities and loading conditions, on pavement stress responses and performance warrants further study.

## Figures and Tables

**Table 1:** Physical properties of materials [14].

	Surface	Base		Base
Type	OGFC	PAC	Type	PC
Asphalt content (%)	4.5	4.9	Cement (kg)	261
Porosity (%)	16.4	17.1	Water (kg)	135
Stability (N)	4446	3329	Porosity (%)	17.6
Retained strength Index (%)	93.5	80.2	7 days compressive strength (kg/cm^2^)	112.8

**Table 2:** Material parameters for each layer of the permeable road pavement model.

Structure Layers	Material	Thickness (m)	Density (kg/m^3^)	Elastic Modulus	Poisson’s Ratio	Coefficient of Friction
Surface layer	OGFC	0.03	1973	6800	0.19	7.02
	PAC	0.10	1958	79	0.28	-
Base layer	PC	0.40	2595	30	0.10	-

**Table 3:** Wheel model dimensions.

Material	Dimension (m)
Diameter	1.074
Loaded radius	0.496
Wheel section width	0.315
Wheel carcass	0.5715

**Table 4:** Wheel model geometry parameters [19].

Geometric Parameters	Density (kg/m^3^)	Elastic Modulus (MPa)	Poisson’s Ratio
Tread and Carcass	719	80	0.3
Rim	8000	200,000	0.3

**Table 5:** Results of *t*-tests.

Depth (cm)	Sample	N	Mean (MPa)	Std. Deviation	Std. Error	*t*	Df	Sig.
3	Measured	40	1.83	2.64	0.42	1.24	74	0.22
	Simulated	40	1.17	2.11	0.33			
8	Measured	40	0.16	0.28	0.04	0.47	78	0.64
	Simulated	40	0.13	0.28	0.04			
13	Measured	40	0.03	0.039	0.006	1.93	72	0.06
	Simulated	40	0.02	0.029	0.005			

## Data Availability

The original contributions presented in the study are included in the article, further inquiries can be directed to the corresponding author.
